# The use of cinacalcet in pregnancy to treat a complex case of parathyroid carcinoma

**DOI:** 10.1530/EDM-14-0056

**Published:** 2014-09-01

**Authors:** K Nadarasa, M Bailey, H Chahal, O Raja, R Bhat, C Gayle, A B Grossman, M R Druce

**Affiliations:** Department of Endocrinology, St Bartholomew's Hospital, London, UK; 1 Imperial Centre for Endocrinology, Imperial College Healthcare NHS Trust, London, UK; 2 Department of Neonatology, King's College Hospital, London, UK; 3 Department of Diabetes, King's College Hospital, London, United Kingdom

## Abstract

We present the case of a patient with metastatic parathyroid carcinoma whose hypercalcaemia was medically managed through two pregnancies. The diagnosis was made when the patient presented with chronic knee pain and radiological findings consistent with a brown tumour, at the age of 30. Her corrected calcium and parathyroid hormone (PTH) levels were significantly elevated. Following localisation studies, a right parathyroidectomy was performed with histology revealing parathyroid carcinoma, adherent to thyroid tissue. Aged 33, following biochemical recurrence of disease, the patient underwent a second operation. A subsequent CT and FDG–PET revealed bibasal pulmonary metastases. Aged 35, the patient was referred to our unit for treatment of persistent hypercalcaemia. The focus of treatment at this time was debulking metastatic disease using radiofrequency ablation. Despite advice to the contrary, the patient conceived twice while taking cinacalcet. Even though there are limited available data regarding the use of cinacalcet in pregnancy, both pregnancies continued to term with the delivery of healthy infants, using intensive medical management for persistent hypercalcaemia.

**Learning points:**

- Parathyroid carcinoma is a rare cause of primary hyperparathyroidism.
- Hypercalcaemia during pregnancy can result in significant complications for both the mother and the foetus.
- The use of high-dose cinacalcet in pregnancy has been shown, in this case, to aid in the management of resistant hypercalcaemia without teratogenicity.

## Background

Parathyroid carcinoma is a rare cause of primary hyperparathyroidism (PHPT) (1). Mortality from this malignancy is normally due to complications related to persistent elevations in serum calcium levels as opposed to tumour burden (1). The management of parathyroid carcinoma is divided into that which is potentially curative, and interventions that aim to control the sequelae of incurable disease (1).

Surgery is the only curative treatment option (2). The medical management of parathyroid carcinoma focuses on control of hypercalcaemia, commonly achieved with the use of volume expansion, occasional loop diuretics and bisphosphonate therapy (1) (2). Unfortunately, the efficacy of these treatments is limited as patients often develop resistance over time. Calcimimetics, such as cinacalcet, have been used to lower serum calcium levels, but there is currently no evidence that they alter the natural course of the disease (1).

The available literature on the safety of therapies in pregnancy for the treatment of PHPT is scarce. This case demonstrates the possibility of safe and effective use of cinacalcet during pregnancy.

## Case presentation

We report a case of a Caucasian patient who was referred to an orthopaedic service at the age of 30 with a 5-year history of intermittent left knee pain. Subsequent radiographs revealed features consistent with a brown tumour in the proximal left tibia. Retrospective history revealed a 2-year history of polyuria, polydipsia, constipation and lethargy. On examination, a 3 cm mass was palpable in the right side of her neck. Biochemistry showed a corrected calcium level of 3.4 mmol/l (normal range, 2.15–2.65) and a PTH level of 53 pmol/l (normal range, 1.6–6.9). Both an ultrasound scan of the parathyroid glands and Technetium-99m sestamibi imaging confirmed an abnormal right upper parathyroid gland. The patient underwent a parathyroidectomy and the histology revealed a 15×12×18 mm parathyroid carcinoma extending to the surgical resection margins, with lymphovascular invasion and adherence to the surrounding thyroid tissue. Post-operative CT and FDG–PET scans showed no evidence of residual disease. Over the following two years, her calcium and PTH levels remained within the reference range. At the age of 33, her calcium and PTH levels began to rise. Repeat ultrasound and sestamibi scans confirmed a recurrence at the site of the primary hyperparathyroidism. She underwent a second operation that included the removal of the right lower parathyroid gland, right lobe of the thyroid and a clearance of the level VI lymph node compartment. Histology confirmed recurrence of parathyroid carcinoma. Three months post-operatively, repeat CT and FDG–PET scans revealed pulmonary metastases. Chemotherapy was discussed although the available data for efficacy are limited and the patient declined this.

## Investigation

The patient was referred to our unit at the age of 35 for management of persistent hypercalcaemia. Biochemistry at this point showed a corrected calcium level of 2.98 mmol/l and a PTH level of 82.7 pmol/l. Cinacalcet was commenced at a low dose and gradually increased to the maximum tolerated dose of 90 mg twice daily. This dose normalised the corrected calcium level to 2.51 mmol/l; however, 4 months later, it had risen to 2.89 mmol/l and zoledronate was infused. A CT scan showed a substantial increase in the pulmonary metastases and a further FDG–PET revealed bony metastatic deposits.

## Treatment

To reduce her serum calcium levels, she underwent radiofrequency ablation to four metastases in the right lung, which successfully normalised her calcium level to 2.37 mmol/l and significantly reduced the PTH level to 28.1 pmol/l. She conceived and was able to stop cinacalcet as the calcium levels were within an acceptable range between 2.60 and 2.99 mmol/l. She required recommencement of cinacalcet 60 mg daily at 16 weeks of gestation when her corrected calcium level reached 3.09 mmol/l despite adequate fluid intake. By 30 weeks of gestation, her calcium level had risen to be persistently above 2.90 mmol/l and the cinacalcet dose was therefore increased to 90 mg daily. Throughout the first pregnancy, the calcium level ranged from 2.60 to 3.39 mmol/l.

Following an uncomplicated delivery, the calcium level had risen to 3.13 mmol/l and the cinacalcet dose was increased to 90 mg twice daily, and the patient also received a bisphosphonate infusion. For ongoing control of the calcium level, she underwent a further radiofrequency ablation to the pulmonary metastases and removal of recurrent parathyroid tissue at level VI, which reduced her calcium level to 2.58 mmol/l and PTH level to 62.4 pmol/l, allowing cinacalcet to be reduced to 30 mg daily. However, a few months later, her calcium level rose again to above 3.00 mmol/l. At this point, the management plan included i.v. fluids, bisphosphonate infusion and a repeat FDG–PET scan to localise any active disease, which may be amenable for further debulking. Before this could be carried out, the patient was found to be pregnant. After detailed counselling, the patient chose to continue with the pregnancy. Cinacalcet was continued with the aim of using the minimal dose. In the first trimester, she could only tolerate a dose of 60 mg daily due to nausea. This maintained her calcium level between 2.69 and 3.21 mmol/l. During the second trimester, the nausea settled allowing the dose of cinacalcet to be increased to 120 mg daily. In the last trimester, the calcium level had risen to 3.30 mmol/l, and she was admitted to hospital for calcium control in advance of her due date and again delivered without any maternal complications. The infant suffered from neonatal hypocalcaemia with the lowest calcium level (1.50 mmol/l) recorded on day 7 *post partum *with a PTH level of <10 ng/l (normal range, 10–70). This was treated with calcium and alfacalcidol. Within 1 month, the serum calcium level normalised to 2.27 mmol/l and both medications were discontinued.

## Outcome and follow-up

Post-delivery, the patient was given a dose of bisphosphonate and was discharged home with a package of care including daily i.v. fluid therapy, regular bisphosphonate injections, ongoing cinacalcet treatment and denosumab (3). However, despite these measures, the calcium level had risen to 4.40 mmol/l. Despite multiple admissions and interventions for lowering calcium level, the patient's health continued to deteriorate and the calcium levels became increasingly difficult to manage. She ultimately succumbed after a prolonged inpatient hospital stay. Both children, now aged 4 and 2, are well with normal development including normal growth to date.

## Discussion

Most patients affected by parathyroid carcinoma will present with symptoms of hypercalcaemia (1) (2). To an extent, hypercalcaemia is ameliorated by the physiological changes in pregnancy (4). Maternal complications occur in over 50% of patients with HPT (5). The rate of foetal complications has been quoted to be as high as 80% (6); however, this can be significantly decreased by appropriate management of gestational HPT as demonstrated in our case (4).

Norman *et al*. (7) carried out a retrospective study investigating the foetal outcome in gestational HPT in 77 pregnancies. The mean calcium level at the time of diagnosis was 2.85 mmol/l (range, 2.65–3.33) (7). Approximately 50% of the pregnancies were lost at 12 weeks (range, 7–23) and 12% of neonates had hypoparathyroidism (7). The study concluded that the foetus is much more likely to be harmed when the serum calcium level reaches 2.85 mmol/l or above (7).

Our patient has undergone surgery twice and the following stage would therefore be medical treatment. Cinacalcet is a novel option for the treatment of gestational HPT and Horjus *et al*. (8) were the first to report its effects on human pregnancy and puerperium.

Cinacalcet was used in a multicentre study involving 29 non-pregnant patients with inoperable metastatic parathyroid carcinoma (9). Over 65% of these patients responded effectively to the treatment (9). A crucial finding from that study was the lack of PTH suppression despite lowering of the serum calcium levels (9). There is no convincing evidence that cinacalcet can alter the course of parathyroid carcinoma, but it can certainly alleviate the hypercalcaemic effects (1) (9).

The use of cinacalcet in pregnant animals has been proven to be safe with no adverse foetal effects observed (10). Pregnant rats were given an oral dose of 50 mg/kg per day (equivalent to a human dose of 720 mg) and this resulted in reduced foetal body weight and reduced maternal food consumption and body weight (10). Pregnant rabbits were given an oral dose of 25 mg/kg per day (equivalent to a human dose of 180 mg) and, although cinacalcet was shown to cross the placental barrier, there were no adverse foetal effects (10). Similar to the study on mice, a decreased maternal food consumption and body weight were observed (10).

Horjus *et al*. (8) successfully demonstrated the safe use of cinacalcet in a pregnant woman with no consequent maternal or foetal complications. Cinacalcet was initiated at 32 weeks of gestation when the maternal corrected serum calcium level was found to be 3.96 mmol/l (8). Cinacalcet was ineffective as a single therapy and therefore calcitonin was added (8). This combined regimen effectively controlled the calcium level during the remaining pregnancy (8).

Although our case is unique, in that it has successfully shown the safe use of cinacalcet as a monotherapy throughout two pregnancies, this medication should still be used with caution in pregnancy. There are only sparse case reports, and the need for randomised validated studies, to prove safety in pregnancy, is imperative.

## Patient consent

Consent not gained as patient has deceased.

## Author contribution statement

The first author is not the named physician of the patient but has permission of the physician who was responsible for the patient. The co-authors have contributed to patient care and finalising the draft.
